# A Multicenter Collaborative Study on the Safety and Efficacy of Laparoscopic Appendectomy During Pregnancy

**DOI:** 10.1002/ags3.70089

**Published:** 2025-09-04

**Authors:** Katsuhiro Ogawa, Tomonori Akagi, Hidefumi Shiroshita, Hiroshi Yoshida, Masashi Miguchi, Ken Eto, Tetsuji Ohyama, Akiyoshi Kanazawa, Keiji Matsuda, Koya Hida, Seiichiro Yamamoto, Takeshi Naitoh, Masafumi Inomata, Japan Society of Laparoscopic Colorectal Surgery

**Affiliations:** ^1^ Department of Gastroenterological and Pediatric Surgery Oita University Faculty of Medicine Oita Japan; ^2^ Department of Gastrointestinal and Hepato‐Biliary‐Pancreatic Surgery Nippon Medical School, Graduate School of Medicine Tokyo Japan; ^3^ Department of Gastroenterological Surgery Hiroshima Prefectural Hospital Hiroshima Japan; ^4^ Department of Surgery The Jikei University School of Medicine Tokyo Japan; ^5^ Biostatistics Center Kurume University Fukuoka Japan; ^6^ Department of Surgery Shimane Prefectural Central Hospital Izumo Japan; ^7^ The Fraternity Memorial Hospital Department of Surgery Tokyo Japan; ^8^ Department of Surgery, Graduate School of Medicine Kyoto University Kyoto Japan; ^9^ Department of Gastroenterological Surgery Tokai University School of Medicine Isehara Kanagawa Japan; ^10^ Department of Lower Gastrointestinal Surgery Kitasato University School of Medicine Sagamihara Japan

**Keywords:** acute appendicitis, fetal loss, laparoscopic appendectomy, open appendectomy, pregnant

## Abstract

**Aim:**

Acute appendicitis in pregnant women requires accurate diagnosis and treatment because of potential pregnancy complications. Laparoscopic appendectomy (LA) has been associated with higher rates of fetal loss than open appendectomy (OA). Therefore, we conducted the first multicenter collaborative analysis in Japan to evaluate the safety and efficacy of LA during pregnancy.

**Methods:**

We retrospectively reviewed 152 pregnant women who underwent appendectomy for acute appendicitis between 2012 and 2021 using data from the Japan Society of Laparoscopic Colorectal Surgery. The patients were divided into the OA (*n* = 92) and LA (*n* = 60) groups. The primary endpoint was fetal loss during hospitalization. Patient demographics, perioperative outcomes, pathology, and pregnancy outcomes were analyzed.

**Results:**

No fetal loss occurred in either group. The operative time (*p* = 0.485) and postoperative complications (*p* = 0.708) were similar between the groups. However, the hospital stay was significantly shorter in the LA group than in the OA group (*p* = 0.037). The OA group had a higher rate of gangrenous or perforated appendicitis (*p* = 0.017).

**Conclusions:**

LA is a safe alternative to OA in pregnant women, with no additional maternal complications and comparable fetal outcomes. LA does not negatively affect fetal health.

## Graphical abstract

Laparoscopic appendectomy (LA) during pregnancy was evaluated in a multicenter retrospective study in Japan. LA showed comparable maternal and fetal safety to open appendectomy (OA), supporting the feasibility and safety of LA in pregnant patients.

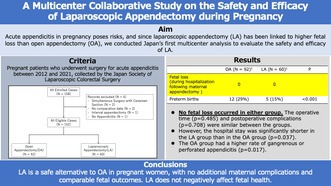

## 1 Introduction

Acute appendicitis during pregnancy tends to progress to a severe condition, and once perforation and subsequent peritonitis occur, the risk of preterm labor or fetal demise increases significantly [1]. Therefore, an accurate diagnosis and timely and appropriate treatment are essential. Several critical factors must be considered when surgical treatment is deemed necessary. These include the potential impact not only on the mother but also on the fetus, limited surgical field owing to the enlarged uterus, and displacement of the appendix in the cephalad and posterior directions, which can make the procedure technically challenging. In open surgery, securing the operative field often requires uterine manipulation or compression, which raises concerns regarding the induction of preterm labor and typically necessitates a large incision.

In contrast, laparoscopic surgery involves smaller incisions and enables a better visual field through the pneumoperitoneum than open surgery, potentially reducing the physical burden on both the mother and fetus. Therefore, this approach is considered advantageous for the continuation of pregnancy and the safety of delivery.

However, a large‐scale meta‐analysis conducted overseas revealed that laparoscopic surgery was associated with a higher fetal mortality rate than open surgery [2, 3, 4]. Based on these findings, the guidelines issued by the Japan Society for Endoscopic Surgery state that “laparoscopic appendectomy in pregnant women was associated with significantly higher rates of miscarriage and fetal mortality than open surgery. The rates of preterm birth and other perioperative or obstetric complications were similar between the groups. Indications for laparoscopic surgery in pregnant women should be carefully considered, and further evaluation through clinical trials is warranted.”

However, in recent years, remarkable advancements have been made in laparoscopic surgical techniques and device development, representing a considerable shift from the context in which the previous reports were generated. Therefore, we aimed to reassess the safety and efficacy of laparoscopic appendectomy (LA) in pregnant women under the current clinical practice in Japan, including its impact on fetal outcomes.

## 2 Methods

### 2.1 Study Design and Setting

This multicenter case–control study was conducted across 35 institutions affiliated with the Japanese Society of Laparoscopic Colorectal Surgery. This retrospective study included pregnant women who had undergone surgery for acute appendicitis between January 2012 and December 2021. The patients were divided into the open appendectomy (OA) and LA groups. The primary endpoint was fetal loss during hospitalization for appendectomy. Patient demographics, perioperative and surgical outcomes, pathological findings, and pregnancy outcomes were analyzed. Preterm delivery was defined as delivery before 37 weeks of gestation and was compared between the OA and LA groups.

Patients were excluded if they met any of the following criteria:

1. Simultaneous surgery with cesarean section, as open surgery was unavoidable in these cases, makes it difficult to compare surgical approaches.
2. Lack of sufficient clinical data for analysis.
3. Interval appendectomy; since these cases were not affected by the acute inflammatory phase and were not suitable for evaluating perioperative and pregnancy outcomes of acute appendicitis.
4. No histopathological confirmation of appendicitis, as these cases were not eligible for inclusion in the study.

Only patients with histologically confirmed acute appendicitis were included in the final analysis.

This study complied with the Declaration of Helsinki and was approved by the Local Institutional Ethics Committee for Clinical Studies in Oita University Hospital (approval number: 2654‐D16). The requirement for written informed consent was waived owing to the retrospective design of the study.

### 2.2 Statistical Analyses

Patient characteristics are presented using the median, interquartile range, and range for continuous variables, as well as the number of cases and percentages for categorical variables. The primary endpoint (fetal loss during hospitalization for appendectomy) was compared between the groups using Fisher's exact test and a logistic regression model adjusted for patient characteristics. Operative time and length of hospital stay were log‐transformed and compared between the groups using multiple linear regression models adjusted for patient characteristics and postoperative pathological severity. Preterm births and pathological findings were compared between the groups using Fisher's exact test. Postoperative complications were compared between the groups using a logistic regression model adjusted for patient characteristics. Missing cases were excluded from the analysis. All *p*‐values were two‐sided, and values < 0.05 were considered statistically significant. All statistical analyses were performed using the R software (version 4.1.1; R Core Team, Vienna, Austria).

## 3 Results

### 3.1 Patient Characteristics

**FIGURE 1:**
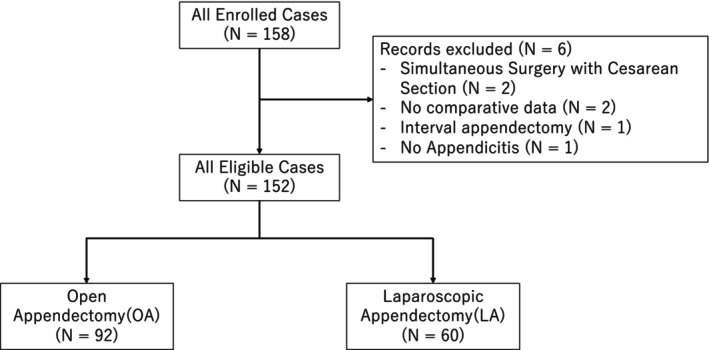
Flow diagram of patient identification and selection.

The patient selection criteria are shown in Figure 1. Data were available for 158 pregnant patients who underwent appendectomy for acute appendicitis, of whom 152 (OA, *n* = 92; LA, *n* = 60) satisfied the study inclusion criteria. The patient characteristics are shown in Table 1. The median ages at the time of surgery were 31 and 30 years in the OA and LA groups, respectively. The median gestational ages at the time of surgery were 20 and 19 weeks in the OA and LA groups, respectively. Pregnancy‐related complications, cardiac disease, respiratory disease, renal disease, and other comorbidities were combined as a single variable in the regression model analysis because they occurred less frequently in the LA group than in the OA group.

**TABLE 1:** Baseline characteristics of patients undergoing OA versus LA during pregnancy.

Variable	OA (*N* = 92)	LA (*N* = 60)
Age at surgery (years)	31.0 (28.0, 34.3) [19.0, 44.0]	30.0 (27.8, 34.3) [19.0, 40.0]
Gestational age at surgery (weeks)	20 (14, 28) [4, 39]	19 (13, 22) [5, 32]
Height (cm)	158.0 (155.0, 161.0) [139.0, 168.0]	160.0 (155.4, 164.0) [147.0, 176.0]
Missing	6	2
Weight (kg)	54 (49, 60) [39, 76]	54 (51, 57) [42, 69]
Missing	4	2
ASA physical status		
I/II	89 (97%)	59 (98%)
III/IV	3 (3.3%)	1 (1.7%)
Pregnancy‐related complications		
None	84 (91%)	58 (97%)
Hypertensive disorders of pregnancy	0 (0%)	0 (0%)
Gestational diabetes	3 (3.3%)	0 (0%)
Other	5 (5.4%)	2 (3.3%)
Cardiac disease	0 (0%)	1 (1.7%)
Respiratory disease	3 (3.3%)	0 (0%)
Renal disease	2 (2.2%)	0 (0%)
Other comorbidities	18 (20%)	4 (6.7%)
History of abdominal surgery	12 (13%)	3 (5.1%)
Missing	0	1

*Note:* Data are presented as the median (interquartile range) [range] or *n* (%).

Abbreviations: ASA, American Society of Anesthesiologists; LA, laparoscopic appendectomy; OA, open appendectomy.

### 3.2 Fetal Outcomes

The primary endpoint of this study, fetal loss after maternal appendectomy, was not observed in the OA or LA groups. No statistical analysis was conducted because no events occurred in either group. Although a lot of data regarding preterm delivery was missing, the incidence of preterm delivery was significantly higher in the OA group than in the LA group (Table 2).

**TABLE 2:** Fetal outcomes after maternal appendectomy.

	OA (*N* = 92)^a^	LA (*N* = 60)^a^	*p*
Fetal loss (during hospitalization following maternal appendectomy)	0	0	
Preterm births	12 (29%)	5 (15%)	< 0.001^b^
Missing	50	27	

Abbreviations: LA, laparoscopic appendectomy; OA, open appendectomy.

^a^ *n* (%).

^b^ Fisher's exact test.

### 3.3 Surgical Outcomes and Complications

In the multiple regression analysis with log‐transformed operative time as the dependent variable, no significant difference in operative time was observed between the OA and LA groups (*p* = 0.485). No cases in the laparoscopic group required conversion to open surgery. Logistic regression analysis showed no significant difference in the likelihood of postoperative complications between the OA and LA groups, with the presence or absence of postoperative complications as the dependent variable (*p* = 0.708). In the multiple regression analysis with log‐transformed length of hospital stay as the dependent variable, the LA group had a significantly shorter hospital stay than the OA group (*p* = 0.037) (Table 3). A statistically significant difference in postoperative pathological findings was observed between the OA and LA groups (*p* = 0.017) (Table 4).

**TABLE 3:** Surgical outcomes and complications.

	OA (*N* = 92)^a^	LA (*N* = 60)^a^	*p*
Operative time (min)	77 (34–255)	60 (17–143)	0.485^b^
Postoperative complications			
Negative	80 (87.0%)	55 (91.7%)	0.708^c^
Positive	12 (13.0%)	5 (8.3%)	
Hospital stay	7.0 (3.0–162.0)	5.5 (2.0–22.0)	0.037^b^

Abbreviations: LA, laparoscopic appendectomy; OA, open appendectomy.

^a^ Median (range); *n* (%).

^b^ *p*‐value from multiple regression analysis including patient characteristics and postoperative pathological severity (gangrenous/perforated vs. others) as covariates.

^c^ *p*‐value from the logistic regression model adjusted for patient characteristics.

**TABLE 4:** Pathological findings.

	OA (*N* = 92)^a^	LA (*N* = 60)^a^	*p*
Pathological findings			
Catarrhal	8 (9.4%)	10 (19%)	0.017
Phlegmonous	36 (42%)	32 (59%)	
Gangrenous	31 (36%)	10 (19%)	
Perforated	10 (12%)	2 (3.7%)	
Unknown	7	6	

Abbreviations: LA, laparoscopic appendectomy; OA, open appendectomy.

^a^ *n* (%).

## 4 Discussion

Acute appendicitis during pregnancy is the most common non‐obstetric surgical emergency. Delays in diagnosis or treatment can lead to severe maternal and fetal complications. Therefore, timely surgical intervention is critical. In this multicenter retrospective study, we found that LA can be safely performed in pregnant patients without increasing maternal or fetal complications compared with OA. Notably, no fetal loss was observed in either group, and the length of hospital stay was significantly shorter in the LA group than in the OA group, supporting the utility of LA in this population.

Several previous reports have raised concerns regarding the safety of LA in pregnant patients. McGory et al. reported a significantly high risk of fetal loss associated with LA, particularly in cases of negative or complicated appendicitis (odds ratio, 2.31) [2]. Wilasrusmee et al. also demonstrated a high risk of fetal loss in their meta‐analysis (risk ratio, 1.91; 95% confidence interval, 1.31–2.77) [3]. However, most of these studies relied on retrospective data from early periods and may have been affected by confounding factors, such as disease severity and surgeon selection bias.

Recent improvements in laparoscopic technology and perioperative management have significantly improved the safety of LA during pregnancy. Cheng et al. analyzed a national health database in Taiwan and reported that LA did not increase the risk of preterm labor, abortion, or cesarean delivery and was associated with short hospital stays [5]. Furthermore, Affleck et al. [6] reviewed 67 cases of LA and cholecystectomy in pregnant women and found no fetal deaths or anomalies.

The safety of laparoscopic cholecystectomy during pregnancy has also been established, supporting its broad application in pregnant patients. Advancements such as low‐pressure pneumoperitoneum, open (Hasson) technique for trocar insertion, adjusted port placement, and tocolytic management have contributed to improved safety [7, 8]. Additionally, modern anesthetic practices effectively minimize the risk of fetal hypoxia or acid–base imbalance during laparoscopy [9, 10].

In our study, a higher proportion of gangrenous or perforated appendicitis was observed in the OA group than in the LA group, suggesting that severe cases tended to be managed with open surgery. This aligns with previous studies showing that the severity of appendicitis is a stronger predictor of adverse fetal outcomes than the surgical approach [2]. This finding may be related to our observation that preterm deliveries were significantly more frequent in the OA group than in the LA group. However, preterm delivery could be influenced not only by the surgical approach for appendicitis but also by other patient‐related factors.

Recently, Sugai et al. conducted a nationwide analysis using the Diagnosis Procedure Combination database in Japan and reported that laparoscopic appendectomy was associated with significantly higher odds of preterm labor, preterm delivery, or abortion in the second and third trimesters compared to open appendectomy (adjusted ORs: 3.37 and 2.57, respectively) [11]. Their study emphasized that the safety profile of LA may vary depending on gestational age and advocated for careful trimester‐specific risk assessment. In contrast, our multicenter clinical study, which utilized institutionally verified clinical data and included gestational timing information, demonstrated no fetal loss in either group and revealed a non‐significant trend toward fewer preterm births in the LA group. These discrepancies may be explained by differences in patient selection, data granularity, surgical expertise, and perioperative protocols. Our findings suggest that with appropriate operative techniques and perioperative management, LA is a safe and feasible option for pregnant patients throughout all trimesters.

Importantly, our data revealed a significantly shorter length of hospital stay in the LA group than in the OA group. After adjusting for patient characteristics and postoperative pathological severity (gangrenous/perforated), the difference in hospital stay between the groups was significant. This suggests that while disease severity may partly explain the longer hospitalization in the OA group, the minimally invasive nature of LA itself likely contributed to shorter recovery times. This finding is consistent with previous reports and highlights the advantages of minimally invasive surgery in promoting rapid maternal recovery and potentially improving perinatal care [4, 5, 12].

Nonetheless, this study has some limitations. The relatively small sample size limited the statistical power to detect rare events, such as fetal loss or major neonatal complications. The retrospective design also introduced potential selection and information biases. There may have been a bias toward severe cases in the OA group (more gangrenous and perforated). Variability in institutional protocols and surgeon experience may have influenced outcomes. Additionally, data regarding preoperative imaging modalities (e.g., ultrasound, MRI, or CT) were not collected, which limits our ability to evaluate diagnostic accuracy. This limitation is underscored by the fact that one case in our cohort was not confirmed as appendicitis on postoperative histopathological examination, highlighting the importance of accurate preoperative diagnosis.

Another limitation is the partial lack of delivery‐related data, which was primarily due to patients choosing to give birth at different institutions. However, follow‐up information on pregnancy outcomes was available for the majority of cases, and no major issues related to loss to follow‐up were identified. This limitation is unlikely to have significantly affected the overall interpretation of maternal or fetal outcomes.

Conducting randomized controlled trials in pregnant patients with acute appendicitis is ethically and logistically challenging. Therefore, well‐designed observational studies, such as the present multicenter analysis, play an important role in informing clinical decision‐making and guiding surgical management in this vulnerable population.

Despite these limitations, our findings provide valuable multicenter evidence that supports the safety and feasibility of LA in pregnant patients. With appropriate surgical expertise and perioperative care, LA may be a viable and even preferable alternative to OA. Further large‐scale prospective studies or national registry‐based investigations are warranted to establish standardized guidelines for the surgical management of appendicitis during pregnancy [13, 14, 15].

This multicenter retrospective study showed that LA can be safely and effectively performed in pregnant patients without increasing maternal or fetal complications compared with OA. LA was associated with a short hospital stay, indicating its potential benefits in terms of enhanced maternal recovery and perinatal management. Although the sample size was limited, the absence of fetal loss and favorable postoperative outcomes in the LA group support its feasibility as a treatment option for appendicitis during pregnancy.

## Author Contributions

**Katsuhiro Ogawa:** methodology, investigation, funding acquisition, writing – original draft. **Tomonori Akagi:** conceptualization, methodology, funding acquisition, writing – review and editing. **Hidefumi Shiroshita:** writing – review and editing, supervision. **Hiroshi Yoshida:** investigation. **Masashi Miguchi:** investigation. **Ken Eto:** investigation. **Tetsuji Ohyama:** validation, formal analysis. **Akiyoshi Kanazawa:** investigation. **Keiji Matsuda:** investigation. **Koya Hida:** investigation. **Seiichiro Yamamoto:** investigation, writing – review and editing. **Takeshi Naitoh:** project administration, supervision. **Masafumi Inomata:** project administration, writing – review and editing, supervision.

## Disclosure

Author Masafumi Inomata is an editorial member of Annals of Gastroenterological Surgery.

## Ethics Statement

This study complied with the Declaration of Helsinki and was approved by the Local Institutional Ethics Committee for Clinical Studies in Oita University Hospital (approval number: 2654‐D16).

## Consent

The authors have nothing to report.

## Conflicts of Interest

The authors declare no conflicts of interest.
